# Human *Polycomb group *EED protein negatively affects HIV-1 assembly and release

**DOI:** 10.1186/1742-4690-4-37

**Published:** 2007-06-04

**Authors:** Dina Rakotobe, Jean-Claude Tardy, Patrice André, Saw See Hong, Jean-Luc Darlix, Pierre Boulanger

**Affiliations:** 1 Laboratoire de Virologie & Pathologie Humaine, Université Lyon I & CNRS FRE-3011, Faculté de Médecine Laennec, 7, rue Guillaume Paradin, 69372 Lyon Cedex 08, France; 2 Laboratoire de Virologie Médicale-Nord, Hôpital de la Croix-Rousse, Hospices Civils de Lyon, 103, Grand'Rue de la Croix-Rousse, 69317 Lyon Cedex 04, France; 3 LaboRétro, Unité de Virologie Humaine, INSERM U-758 & IFR128 BioSciences Lyon-Gerland, Ecole Normale Supérieure, 46, allée d'Italie, 69364 Lyon Cedex 07, France; 4 Laboratoire de Virologie Médicale, Hospices Civils de Lyon, CBPE, 59, Boulevard Pinel, 69677 Bron Cedex, France

## Abstract

**Background:**

The human EED protein, a member of the superfamily of *Polycomb *group (*Pc*G) proteins with WD-40 repeats, has been found to interact with three HIV-1 components, namely the structural Gag matrix protein (MA), the integrase enzyme (IN) and the Nef protein. The aim of the present study was to analyze the possible biological role of EED in HIV-1 replication, using the HIV-1-based vector HIV-Luc and EED protein expressed by DNA transfection of 293T cells.

**Results:**

During the early phase of HIV-1 infection, a slight negative effect on virus infectivity occurred in EED-expressing cells, which appeared to be dependent on EED-MA interaction. At late times post infection, EED caused an important reduction of virus production, from 20- to 25-fold as determined by CAp24 immunoassay, to 10- to 80-fold based on genomic RNA levels, and this decrease was not due to a reduction of Gag protein synthesis. Coexpression of WTNef, or the non-N-myristoylated mutant NefG2A, restored virus yields to levels obtained in the absence of exogenous EED protein. This effect was not observed with mutant NefΔ57 mimicking the Nef core, or with the lipid raft-retargeted fusion protein LAT-Nef. LAT_AA_-Nef, a mutant defective in the lipid raft addressing function, had the same anti-EED effect as WTNef. Cell fractionation and confocal imaging showed that, in the absence of Nef, EED mainly localized in membrane domains different from the lipid rafts. Upon co-expression with WTNef, NefG2A or LAT_AA_-Nef, but not with NefΔ57 or LAT-Nef, EED was found to relocate into an insoluble fraction along with Nef protein. Electron microscopy of HIV-Luc producer cells overexpressing EED showed significant less virus budding at the cell surface compared to control cells, and ectopic assembly and clustering of nuclear pore complexes within the cytoplasm.

**Conclusion:**

Our data suggested that EED exerted an antiviral activity at the late stage of HIV-1 replication, which included genomic RNA packaging and virus assembly, resulting possibly from a mistrafficking of viral genomic RNA (gRNA) or gRNA/Gag complex. Nef reversed the EED negative effect on virus production, a function which required the integrity of the Nef N-terminal domain, but not its N-myristoyl group. The antagonistic effect of Nef correlated with a cellular redistribution of both EED and Nef.

## Background

EED protein, the human ortholog of the mouse embryonic ectoderm development (*eed*) gene product, is a member of the superfamily of WD-40 repeat proteins and widely conserved *Polycomb *group (*Pc*G) family of proteins [1-7]. The human EED protein, also called WAIT-1 (for WD protein associated with integrin cytoplasmic tails-1; [8]), can interact with the cytoplasmic tail of integrin β7 subunit, a domain which is involved in major integrin functions such as receptor affinity and signaling [9,10]. EED was also found to interact with three HIV-1 proteins, the Gag matrix protein MA [11], the integrase enzyme IN [12] and the Nef regulatory protein [13]. Although recognized as a nuclear factor, EED has been shown to shuttle between the nucleus and the plasma membrane [8], where it forms a complex with HIV-1 Nef releasing an EED-mediated transcriptional block [13]. The data obtained with Nef and EED were consistent with the known functions of *Pc*G proteins, which participate in the maintenance of the silent state of chromatin in upper eukaryotes, such as in female X chromosome inactivation [14], and generally act as transcriptional repressors of homeotic genes (reviewed in [15-18]). They were also consistent with the finding that HIV-1 preferentially integrates into transcriptionally active regions of the host genome [19-22]. Thus, regions of cellular genome unoccupied by EED or EED-containing multiprotein complexes might be preferred targets for proviral DNA integration.

EED is part of multiprotein edifices called Polycomb Repressive Complexes (PRCs) that are found in *Drosophila *and in mammals [17]. Several types of PRCs have been identified and commonly called PRC1, PRC2 and PRC3 [23]. PRC2/3 contain at least five components, EED, EZH2, SUZ12, RbAp38 and AEBP2 [23-25]. Four isoforms of human EED have been identified [24], due to alternative translation initiations at codons specific for Val1 (EED1), Val36 (EED2), Met95 (EED3) and Met110 (EED4), respectively, as aligned with the mouse EED sequence of 535 residues [5,7], and not to alternative splicing of the *eed *transcript, as previously hypothesized [11]. It is generally accepted that PRC3 complex contains the two shorter forms of EED (EED3, EED4), while PRC2 contains the longer EED1 form, and the intermediate EED2 form is present in another distinct PRC complex [23]. However, a more dynamic and flexible view of the PRC composition has been proposed [17].

Because EED can interact with three major HIV-1 components, we wanted to investigate the interplay between EED and the virus in infected cells. We found that EED isoforms 3 and 4 (EED3/4) had only a moderate antiviral activity on infecting virions, whereas at the late phase of virus replication, EED3/4 showed a strong negative effect on virus production. Interestingly, this effect was reversed by WTNef, and its non-N-myristoylated mutant NefG2A, implying that it was not dependent on Nef packaging into virions. No anti-EED effect was observed with the N-terminal deletion mutant called NefΔ57, or with LAT-Nef, a Nef fusion protein targeted to the membrane microdomains known as lipid rafts [26]. The EED antagonistic function of Nef was associated with a cellular redistribution of EED3/4 proteins, whereby EED and Nef were depleted from the membranes and redirected to a still undefined compartment. EED did not inhibit Gag protein synthesis, and our results suggested that virus assembly and genome packaging were the major targets of the EED inhibitory activity.

## Results

### Effect of EED3/4 on incoming HIV-1

The observation that isoforms 3 and 4 of EED were recovered in the same PRC3 complex [23] suggested that certain biological functions probably required the EED3-EED4 pair. In the HIV-1 context, we found that the MA protein interacted with EED via a single site common to shorter and longer isoforms [11], and that the IN bound to EED via two discrete regions contained within residues 95–535, corresponding to EED3 [12]. We therefore kept the Met-codon at position 110, which could function as a natural alternative initiator of translation, allowing the simultaneous expression of both EED3 (441 residues) and EED4 (428 residues) isoforms, abbreviated EED3/4 in the present study.

**Figure 1:**
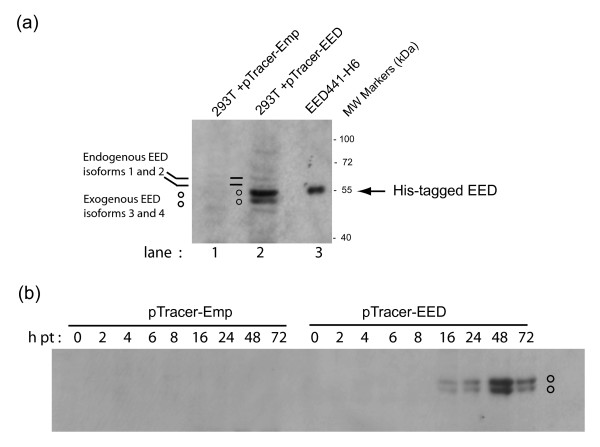
**Over-expression of EED3/4 isoforms in 293T cells**. **(a)**, SDS-PAGE and radioimmunoblot analysis of soluble fraction of 293T cell lysates after transfection with pTracer-Emp (lane 1) or pTracer-EED (lane 2) ; bacterially-expressed, histidine-tagged isoform 3 (EED441-H6; arrow) is shown in lane 3. **(b)**, Kinetics of transient expression of exogenous EED3/4, using pTracer-EED versus control empty plasmid pTracer-Emp. Autoradiograms of blots reacted with anti-EED antibody and ^35^SLR-labeled anti-rabbit IgG antibody. Note that the endogenous EED proteins were barely detectable in soluble lysates from 293T cells, whereas exogenous EEDs were visible as a doublet band at 52 and 51 kDa, detectable as early as 16 h after transfection with a maximum at 48 h.

In whole cell lysates from control 293T cells (Fig. 1a, lane 1 ; Fig. 1b, left half of the panel), only trace amounts of endogenous EED were detected. In 293T cells transfected with pTracer-EED, the expected doublet band corresponding to exogenous EED3 and EED4 proteins at 52 and 51 kDa, respectively, was observed (Fig. 1a, lane 2). In kinetics analysis, EED3/4 proteins were clearly accumulating at 16 h, with a maximum level at 48 h (Fig. 1b; lanes 16 h to 72 h).

**Figure 2:**
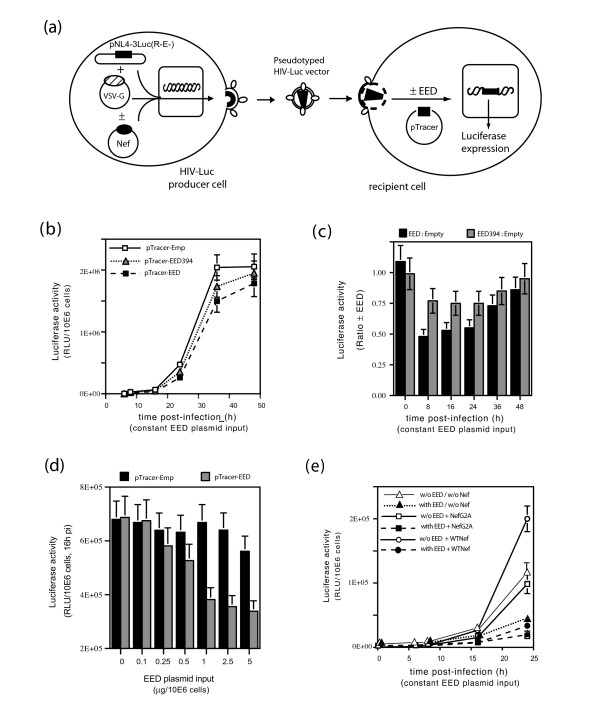
**Antiviral effect of EED3/4 on incoming HIV-Luc virions**. **(a), **Experimental protocol. **(b), **Time-course analysis of luciferase expression in 293T cells transfected with pTracer-EED, pTracer-EED394 (EED ST394AI mutant) or control pTracer-Emp at constant plasmid input (1 μg/10^6 ^cells), and infected with HIV-Luc vector. Luciferase activity, expressed as relative light units (RLU), was normalized to equal protein content. **(c), **Ratio of luciferase levels in cells expressing EED3/4 or EED394, versus control (pTracer-Emp). **(d), **Dose-response analysis of EED3/4 effect on luciferase expression.**(e), **Effect of the coexpression of WTNef or packaging-defective mutant NefG2A on EED antiviral activity. The discrete negative effect of EED on virus infectivity did not change when NefWT was provided in *trans *to virions in producer cells.

To determine the possible effect of EED on incoming HIV-Luc virions in a single-round replication assay, 293T cells expressing EED3/4 proteins were infected by VSV-G-pseudotyped HIV-Luc, and luciferase expression assayed at different times post-infection (pi) and at different pTracer-EED inputs (Fig. 2a). The HIV-driven luciferase activity was found to be at modestly but consistently lower levels in EED3/4-expressing cells compared to control cells (Fig. 2b, c), with a maximum effect (2–3-fold) observed at 8 to 24 h pi. The negative effect of EED3/4 on HIV-Luc expression occurred in a dose-dependent manner (Fig. 2d), and was less pronounced with the MA-binding defective mutant EED394 (Fig. 2b, c), suggesting that this depended, at least in part, on EED-MA interaction.

Because the *luc *gene has been inserted into the *nef *region of HIV-Luc genome, the HIV-1 virions used in the above experiments lacked the Nef protein [27] shown to be an EED interactor [13]. We then expressed the Nef protein in *trans *in HIV-Luc-producer cells (Fig. 2a), and examined whether Nef incorporation into virions could overcome the negative effect of exogenous EED3/4. As expected from previous studies [28], WTNef increased the infectivity of HIV-Luc by a factor of 2-fold at all time points, compared to vector produced in the absence of Nef or in the presence of the packaging-defective mutant NefG2A (Fig. 2e). However, the packaging-competent WTNef did not change the negative effect of EED3/4 on HIV-Luc expression in newly infected cells (Fig. 2e).

### Effect of EED3/4 on virion production

**Figure 3:**
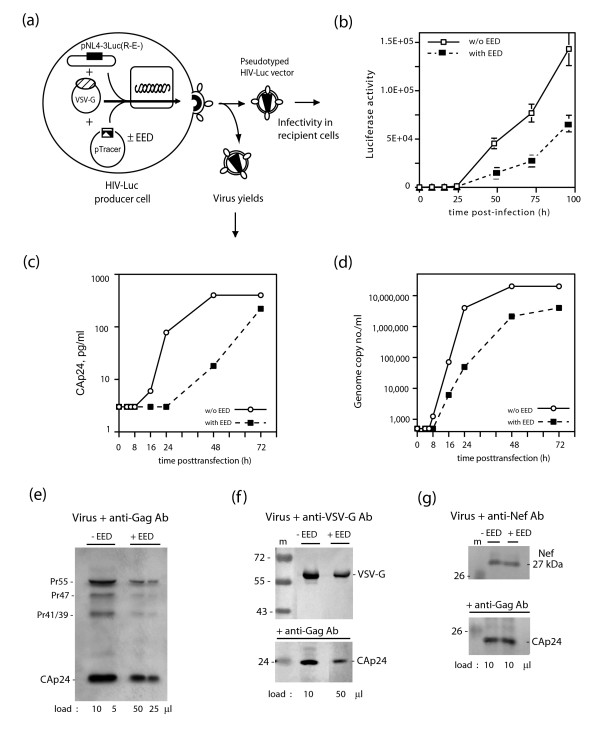
**Influence of EED3/4 expression on virus yields**. **(a), **Experimental protocol. **(b), **Virus infectivity. Virions produced by 293T cells transfected with pTracer-EED (filled symbols) or control pTracer-Emp plasmid (open symbols) were used to infect recipient 293T cells, and luciferase expression monitored at different times pi, as indicated. **(c, d), **Vector titer was determined by CAp24 immunoassay (c) or genomic RNA level (d). **(e-g)**, SDS-PAGE polypeptide pattern of virus particles released from cells transfected with pTracer-EED (+EED) or control pTracer-Emp plasmid (-EED). Blots were reacted with anti-Gag (e; f, bottom panel ; g, bottom panel), anti-VSV-G (f, top panel) and anti-Nef (g, top panel) antibodies. Virus production was significantly reduced in the presence of EED, ranging from 20- to 25-fold at 24–48 h posttransfection, based on CAp24 immunoassay (c), to 10- to 80-fold, based on genomic RNA levels (d). The Gag protein composition (e), the VSV-G-to-CAp24 (f) and the Nef-to-CAp24 (g) ratios did not differ significantly between particles produced in the presence or absence of EED. Note that the load of virus samples produced in the presence of EED (e ; +EED) was 5-fold higher than control samples (-EED), and that coexpression of Nef restored the virus yields, as shown by CAp24 immunoblotting (g ; bottom panel).

The influence of EED3/4 on virus production and infectivity was investigated as illustrated in Fig. 3a, using cells cotransfected with pNL4-3Luc(R-E-), phCMV-G encoding VSV-G and pTracer-EED or pTracer-Emp ('empty vector DNA' used as control). Cell culture supernatants were harvested 48 h after DNA transfection, and virions recovered and purified as described in Materials & Methods. Virion-containing fractions were extensively characterized with respect to virus infectivity based on luciferase activity in recipient cells, and levels of virion genomic RNA, and RT activity (Fig. 3a). Luciferase activity was reduced by about 4- to 5-fold at 96 h pi when HIV-Luc was produced by EED3/4-expressing cells, compared to control cells (Fig. 3b).

Virion production, as monitored by CAp24-ELISA, was found to be lower from EED3/4-cells, in comparison with control cells (about 20-fold lower at 48 h posttransfection; Fig. 3c). Likewise, the level of virion genomic RNA was strongly diminished (at least 30-fold less) in particles produced by EED3/4-expressing cells in comparison with control samples at 16 to 48 h posttransfection (Fig. 3d). The mean density value and Gag protein composition of virions did not change upon EED expression in HIV-1 producer cells (Fig. 3e). Virions were also probed for possible copackaging of EED, but no detectable EED3 or EED4 protein was found in vector particles (not shown). The lower infectivity of virus samples yielded by EED3/4-expressing cells was not due to a lower level of cellular expression and viral incorporation of VSV-G (and Nef, when Nef was co-expressed in the same cell ; refer to Figure 6), as the ratios of virus-encapsidated VSV-G to CAp24 and Nef to CAp24 were not significantly different in the presence or absence of EED3/4 (Fig. 3f and 3g).

We quantitated the EED3+EED4 and Gag contents in plasmid-transfected cells, and found that the whole cell content was in the range of 10^6 ^EED and 10^7 ^Pr55Gag molecules per cell at 48 h posttransfection with 1 μg of pTracer-EED and pNL4-3Luc(R-E-), i.e. a EED:Gag ratio of 1:10. The endogenous EED3+EED4 protein content was estimated to be ca. 20 to 30 times less, i.e. 3 × 10^4 ^to 5 × 10^4 ^EED per cell. Considering that the core of a mature virion is constituted of 1,400–1,500 Gag molecules [29,30], we calculated a ratio of 150 molecules of exogenous EED3/4 per assembling virion in HIV-producing cells.

**Figure 4:**
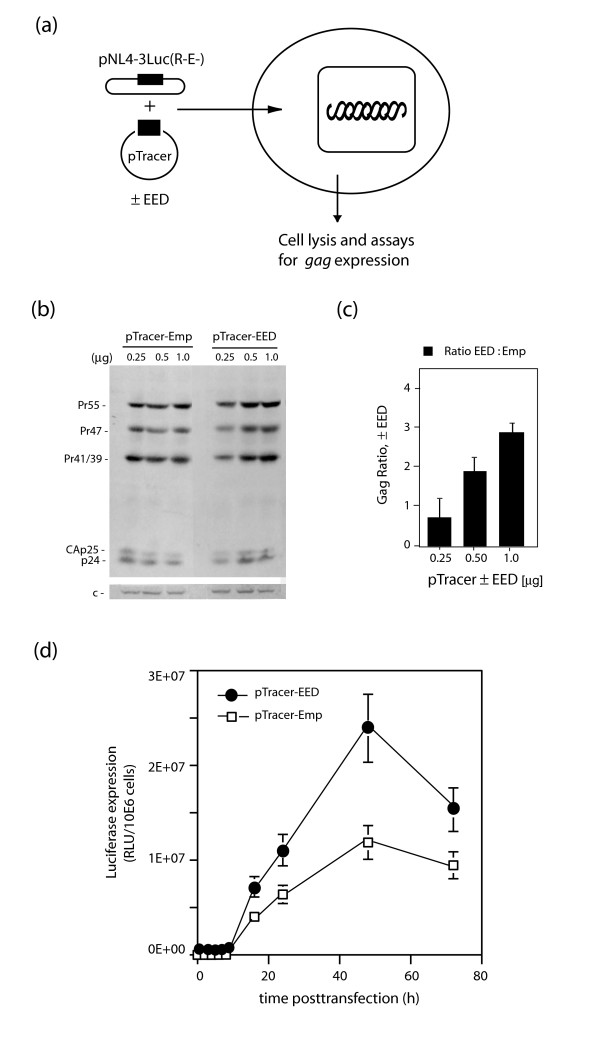
**Gag protein expression in EED3/4-expressing cells**. **(a), **Experimental protocol. **(b, c), **Dose-response analysis of EED3/4 effect on *gag *gene expression in cells cotransfected with pNL4-3Luc(R-E-) and pTracer-EED (or control pTracer-Emp), at varying plasmid inputs (0.25 to 1 μg/10^6 ^cells). **(b), **Autoradiogram of SDS-PAGE and immunoblots reacted with anti-Gag antibody and ^35^SLR-labeled complementary antibody. Band c, 20-kDa cellular protein used as internal control for protein load. **(c), **Histogram of the ratios of total Gag proteins synthesized in the presence of pTracer-EED versus pTracer-Emp. **(d), **Time-course analysis of pNL4-3Luc(R-E-)-mediated luciferase expression in 293T cells co-transfected with pTracer-EED (filled symbols) or control pTracer-Emp plasmid (open symbols) at constant plasmid input (1 μg/10^6 ^cells). A slight increase in Gag protein synthesis was detected in the presence of EED, at plasmid inputs higher than 0.5 μg. A similar positive effect of EED (2- to 5-fold) on luciferase levels was observed between 18 and 72 h posttransfection.

We next examined whether the negative effect of EED on virion production reflected or not a lower level of Gag precursor synthesis. Cells were cotransfected with a constant amount of pNL4-3Luc(R-E-) and increasing amounts of either the pTracer-EED or pTracer-Emp plasmid (Fig. 4a). Gag synthesis was monitored by western blotting showing the different forms of Gag protein, namely Pr55Gag, the partially processed products Pr47Gag and Pr41/39, and CAp25/CAp24. Gag proteins were present in all conditions, and at consistently higher plateau levels in EED3/4-expressing cells compared to control cells lacking exogenous EED3/4 (Fig. 4b). The EED positive effect on Gag synthesis occurred in a dose-dependent manner, with a range of 1.5- to 3-fold for 0.5 to 1 μg of the EED-expressing plasmid (Fig. 4c). A similar level of enhancement (2- to 4-fold with 1 μg of EED plasmid) was observed for luciferase activity in the presence of EED3/4 (Fig. 4d). This indicated that the negative effect of EED3/4 on virion production was not caused by the down-regulation of *gag *expression, but probably due to a defect in Gag assembly and virus production.

### Interfering RNA targeting EED increases virion production

**Figure 5:**
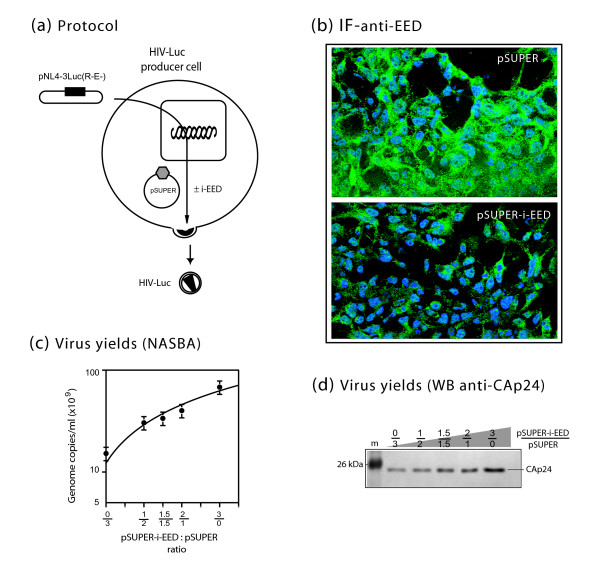
**RNA interference targeting endogenous EED**. **(a), **Experimental protocol. 293T cells were transfected with a constant amount (3 μg/10^6^cells) of a mixture of pSUPER + pSUPER-i-EED at various ratios of each plasmid, and posttransfected with pNL4-3Luc(R-E-) 24 h later. Virus yields were determined in culture medium after a further 48 h incubation. **(b) **Immunofluorescence (IF) signal of endogenous EED proteins in cells transfected with control pSUPER (upper panel) or pSUPER-i-EED (lower panel) at 3 μg/10^6^cells each, and reacted with anti-EED antibody (1:200) and FITC-labeled conjugate(1:320). **(c, d), **Virion production was monitored by genomic RNA levels **(c), **and CAp24 immunoassays **(d)**. Virus production increased in parallel with the decrease of EED signal and as a function of pSUPER-i-EED input, with a maximum of 4- to 5-fold over the control.

To determine whether inhibition of endogenous EED expression would affect virion production, cells were transfected with a constant amount of a mixture of pSUPER and pSUPER-i-EED at varying ratios of the two plasmids. One day later, cells were transfected with pNL4-3Luc(R-E-) and virion levels determined after a further 48 h-incubation period (Fig. 5a). Since endogenous EED isoforms were barely seen on immunoblots (refer to Fig. 1), the inhibition of EED protein synthesis by pSUPER-i-EED was monitored *in situ *by immunofluorescence of transfected cells using anti-EED antibody (Fig. 5b). The amount of virions produced increased as a function of the quantity of transfected pSUPER-i-EED and in parallel with the decrease of the EED signal (Fig. 5b), to reach a maximum of 4- to 5-fold over the control (Fig. 5c, d). These results confirmed that EED has a negative effect on virion production.

### Nef antagonizes the negative effect of EED on virus production and genome encapsidation

**Figure 6:**
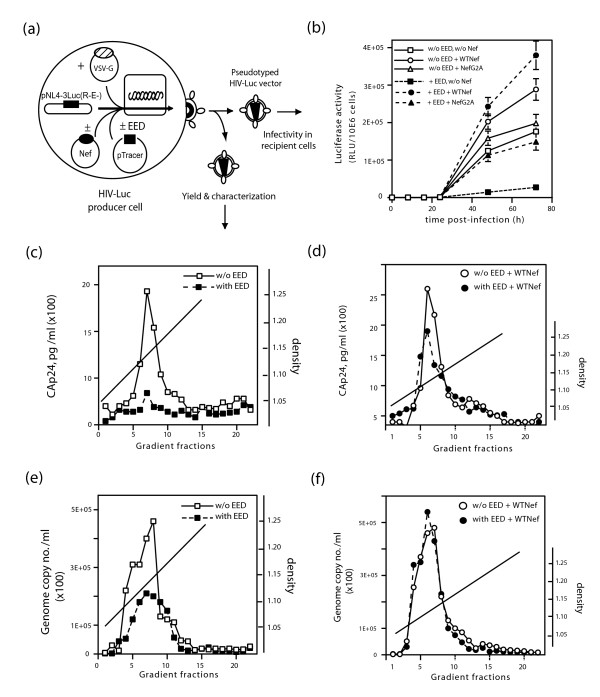
**Antagonistic effect of Nef on EED**. **(a), **Experimental protocol. Pelleted HIV-Luc vector particles produced by 293T cells transfected with pTracer-EED (filled symbols) or pTracer-Emp (open symbols), with or without Nef protein, WTNef or NefG2A mutant, were assayed for **(b) **vector infectivity, determined by luciferase activity in recipient cells, or **(c-f) **further analyzed by sucrose-D_2_O gradient ultracentrifugation. Gradient fractions were analyzed for **(c, d) **CAp24 titer, and **(e, f) **genomic RNA level. WTNef protein counteracted the negative effect of EED, and restored the virus production to control levels.

Since Nef forms a complex with EED at the plasma membrane of HIV-1-infected cells, thus contributing to EED nuclear depletion [13], we wanted to examine the influence of Nef on the anti-viral activity of EED3/4 (Fig. 6a). WTNef restored the HIV-Luc infectivity to a level similar to that obtained in the absence of EED3/4 (Fig. 6b). The N-myristoylation-negative, packaging-defective mutant NefG2A showed no significant effect on virus infectivity in the absence of EED3/4, but when coexpressed with EED3/4, NefG2A partly restored virus infectivity (Fig. 6b). This implied that the membrane targeting and encapsidation of Nef were not absolute prerequisites for the Nef antagonistic effect. The deletion mutant NefΔ57, representing the Nef core [31,32], showed no EED-counteracting activity (not shown), confirming the assignment of residues 16–35 within the N-terminal domain of Nef as one the two EED-interacting sites [13].

The production of virions made in the presence Nef and EED3/4 and their genomic RNA content confirmed the antagonistic effect of Nef on EED (Fig. 6c–f). With EED and WTNef, the virion yields were similar to the levels obtained in the absence of EED3/4 (Fig. 3g and Fig. 6c, d), and the genome copy number was even slightly higher (Fig. 6e, f). Of note, the ratio of genome copy number to virion CAp24 was consistently higher (35–40 %) in virions produced in the presence of both EED3/4 and WTNef than that in the presence of WTNef alone, namely 2.86 ± 0.19 × 10^4 ^versus 1.87 ± 0.21 × 10^4 ^genome copies/pg CAp24 (m ± SD, n = 4), respectively.

**Figure 7:**
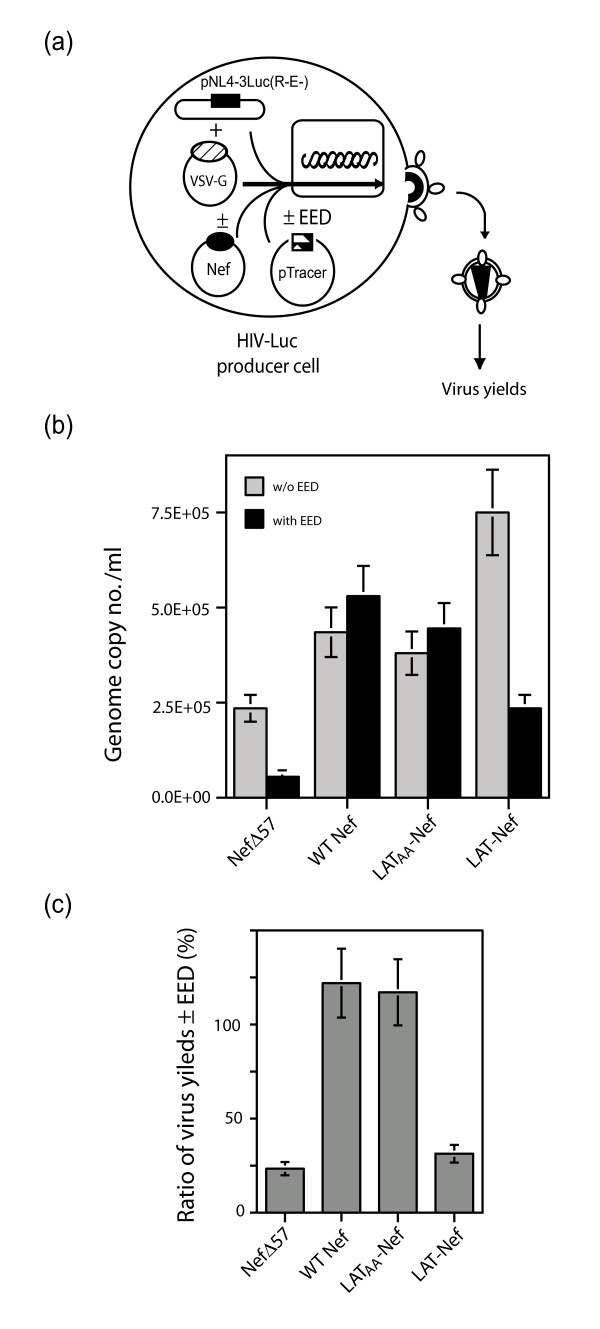
**Effect of Nef mutants on EED**. **(a), **Experimental protocol. HIV-Luc virions were produced by 293T cells transfected with pTracer-EED or pTracer-Emp, in the presence of various Nef proteins, WTNef, NefΔ57 mutant, or fusion constructs LAT-Nef or LAT_AA_-Nef. Cell culture fluids were centrifuged and the amounts of virions in pellets quantified by genomic RNA levels. **(b), **Histogram of virion production in the presence of Nef without EED (grey bars) or EED with Nef (filled bars). **(c), **Ratio of virus yields in the presence versus the absence of EED, expressed as percentage. WTNef and LAT_AA_-Nef showed the same EED antagonistic effect, whereas LAT-Nef and NefΔ57 mutants had a different phenotype.

To further dissect the antagonistic effect of Nef on EED, two other forms of Nef were used, referred to as LAT-Nef and LAT_AA_Nef mutant, respectively [26]. Both were fusion proteins carrying the first 35 amino acids of the linker of the activated T-cell factor (LAT) at the N-terminus of the full-length Nef sequence. LAT_AA_Nef differed from LAT-Nef by the substitution of the cysteines 26 and 29, which are palmitoylated, to alanine. Addition of the LAT sequence was designed in order to target all Nef molecules to lipid rafts, while LAT_AA_Nef protein served as the control due to its cytosolic localization [26]. LAT-Nef showed no EED antagonistic effect, whereas LAT_AA_Nef behaved as WTNef in terms of virion yields (Fig. 7). This confirmed that membrane targeting was not required for the anti-EED function of Nef, and suggested that the anti-EED function of Nef involved a subset of Nef molecules which were not localized in the lipid rafts. The next experiments examined whether the negative effect of EED on Gag assembly and the antagonistic effect of WTNef, NefG2A and LAT_AA_Nef proteins on EED were associated with alterations of protein compartmentalization.

### Influence of Nef on the cellular distribution of EED

**Figure 8:**
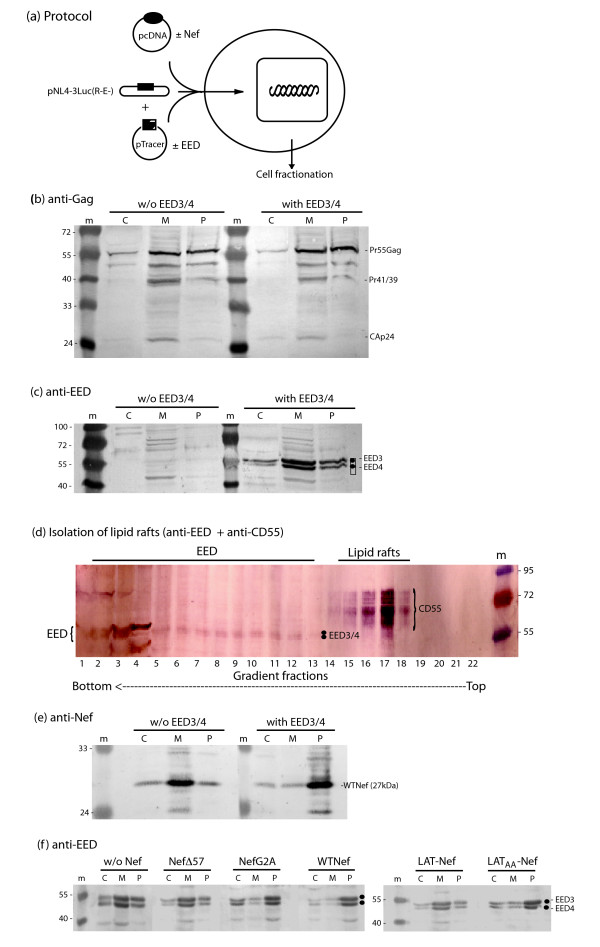
**Cellular distribution of EED3/4 upon NEF expression**. **(a), **Experimental protocol. Cells were cotransfected with pNL4-3Luc(R-E-) and pTracer-EED (or pTracer-Emp), with or without coexpression of various Nef proteins, WTNef or NefG2A and NefΔ57 mutants, or fusion constructs LAT-Nef or LAT_AA_-Nef, as indicated on top of each panel. Cells were fractionated into cytosolic supernatant (C), membrane fraction (M) and insoluble pellet (P), as shown in panels **(b)**,**(c)**,**(e) **and **(f). **Fractions were probed for **(b) **Gag, **(c, d, f) **EED, **(e) **Nef, and **(d) **CD55. **(d), **Isolation of lipid rafts by ultracentrifugation of flotation. Gradient fractions were analyzed by SDS-PAGE and immunoblotting, using anti-CD55 antibodies (detected by phosphatase-labeled complementary antibody) and anti-EED antibodies (detected by peroxidase-labeled complementary antibody). (m), Protein markers, with molecular masses indicated in kDa. Bands of exogenous EED3 and EED4 isoforms are indicated by black dots. Note that EED did not cosediment with lipid rafts, identified by the CD55 marker. Coexpression of EED and WTNef, NefG2A or LAT_AA_-Nef, but not NefΔ57 or LAT-Nef, resulted in the relocation of EED and Nef proteins in a cellular compartment recovered as pelletable fraction (P).

Cells cotransfected with pNL4-3Luc(R-E-), pTracer-EED (or pTracer-Emp), with or without Nef, were fractionated into cytosolic fraction (C), membrane compartment (M) and insoluble pellet (P) (Fig. 8a), and each fraction probed for Gag, EED and Nef proteins. As expected, Gag polyprotein precursor and maturation products were mainly detected in fractions M and P, and in small amounts in cytosol (Fig. 8b ; left panel). Expression of EED3/4 did not significantly change the distribution of Gag between the three compartments (Fig. 8b ; right panel). Exogenous EED3/4 proteins were found in all three compartments, with a predominance in the membrane fraction M (Fig. 8c, right panel), as were the endogenous EED's (Fig. 8c, left panel). Further fractionation of the M compartment showed that the membrane domains where EED localized were different from the lipid rafts (Fig. 8d). WTNef protein was recovered in majority in fraction M, but upon EED3/4 coexpression, we observed an apparent Nef depletion from the M compartment and its relocation to the insoluble fraction P (Fig. 8e). The same change in the EED pattern was observed in the presence of WTNef, with relocation of EED to fraction P (Fig. 8f). EED relocation in fraction P also occurred in the presence of NefG2A or LAT_AA_Nef, but not with the deletion mutant NefΔ57 (cytosolic) or LAT-Nef (lipid raft-targeted) (Fig. 8f).

**Figure 9:**
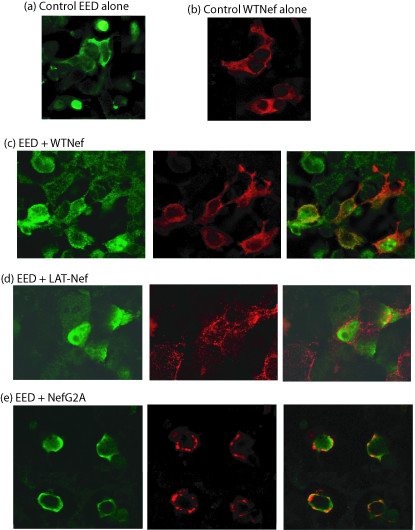
**Confocal fluorescence microscopy of 293T cells expressing EED alone **(a) **or WTNef alone **(b), **or co-expressing EED and various Nef proteins; **(c),**WTNef ;**(d),**LAT-Nef ;**(e), **NefG2A.** The experimental protocol was as described in Fig. 8a. Left panels: anti-EED rabbit antibody and Alexa Fluor^® ^488-labeled goat anti-rabbit IgG ; middle panels: anti-Nef mAb and Alexa Fluor^® ^633-labeled goat anti-mouse IgG antibody; right panels: merged images. Note the absence of co-localization of EED and LAT-Nef, contrasting with the co-localization signals of EED and WTNef or NefG2A.

Immunofluorescence (IF) analysis confirmed the cell fractionation patterns, and showed the absence of co-localization of EED and LAT-Nef proteins. On the contrary, the EED and WTNef signals co-localized in the cytoplasmic compartment (Fig. 9). Likewise, co-localization occurred for EED and NefG2A proteins, and the IF pattern suggested that they co-localized in large intracytoplasmic inclusions (Fig. 9e).

#### Electron microscopy

**Figure 10:**
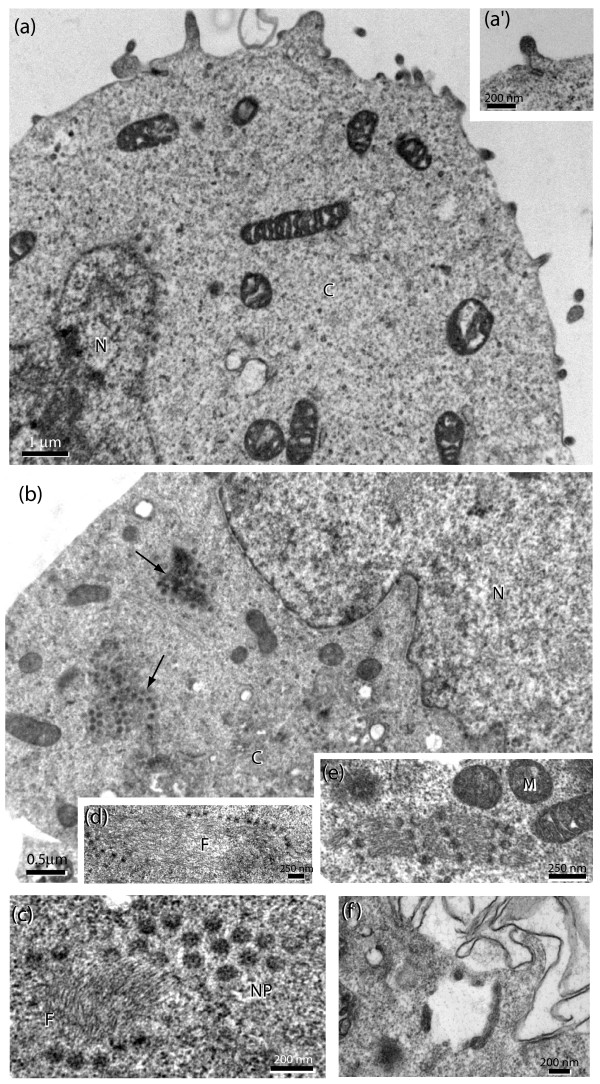
**Electron microscopic analysis of 293T cells cotransfected with pNL4-3Luc(R-E-) and pTracer-Emp (**a**, and inset **a'**) or pTracer-EED (**b-f**) at 3 μg each plasmid per 2 × 10^6 ^cell sample, and harvested at 48 h posttransfection**. **(a), **Control cells without exogenous EED expression. Note the number of viral particles budding at the cell surface. Inset **(a')**, Enlargement of one virus particle at an intermediate step of budding and egress. **(b), **EED3/4-expressing cells showing very rare budding events at the plasma membrane. Several clusters of ringlike structures (arrows) were observed in the cytoplasm, at distance from the nuclear envelope. Their dimensions (70–80 nm in overall diameter) and constitutive elements (electron-dense annular granules of 15–18 nm in diameter and central channel of 25–27 nm) were characteristic of nuclear pore complexes. **(c-f), **Enlargement of cytoplasmic areas from EED3/4-expressing cells showing clusters of nuclear pores (NP), viewed in tangential **(c) **or transversal **(d, e) **section, and in association with filaments arranged in bundles (F). **(f), **Cytoplasmic area of EED3/4-expressing cell showing intracytoplasmic vesicles at higher magnification. Note the local thickening of the vesicular membrane and the intraluminal budding of virus-like particles. N, nucleus ; C, cytoplasm ; M, mitochondria.

Electron microscopic analyses were carried out using 293T cells cotransfected with pNL4-3Luc(R-E-) and pTracer-Emp (Fig. 10a, and inset a') or pTracer-EED (Fig. 10 b–f). A number of HIV-1 virion particles were seen budding at the surface of control cells (Fig. 10, see inset a' where an intermediate step of budding and egress was observed). Upon EED3/4 expression, only rare cells exhibited budding events at the plasma membrane. Interestingly, several clusters of nuclear pore complexes were found in the cytoplasm, at distance from the nuclear envelope (Fig. 10b; arrows). Enlargement of cytoplasmic areas from EED3/4-expressing cells showed clusters of nuclear pore complexes viewed in tangential (Fig. 10b, c) or transversal section (Fig. 10d, e), and associated with bundles of filaments [see Additional files 1 and 2]. The cytoplasmic compartment of EED3/4-expressing cells also showed an abundant vesicular network. At higher magnification, we frequently observed a local thickening of the vesicular membrane and irregular protrusions into the lumen, reminiscent of intracisternal budding of virus or virus-like particles (Fig. 10f). These results further confirmed that EED impacted on HIV-1 assembly and release.

## Discussion

In the present study, we found that overexpression of human EED3/4, a member of the *Pc*G proteins, resulted in a global anti-HIV-1 effect. At early steps of infection, EED3/4 had a modest negative impact on incoming HIV-Luc virions (2- to 3-fold at best; Fig. 2), in comparison with that caused by known cellular restriction factors [33-36]. The limited negative effect caused by EED394, a mutant defective in MA binding [11], suggested that this relied on the integrity of EED-MA interaction. At the late phase of the virus life cycle, EED exerted a significant negative effect on virion production by EED3/4-expressing cells, with 10- to 80-fold lower virus yields (Fig. 3). This effect was not due to an EED-mediated negative effect on Gag protein synthesis, since higher levels of Gag protein, as well as the reporter gene product luciferase, were produced by EED-expressing cells (Fig. 4).

Quantification of the intracellular content of EED and Gag showed a ratio of 1 to 10 in terms of EED3/4 to Gag proteins, which corresponded to approximately 150 copies of EED3/4 proteins available per virus particle containing 1,500 Gag molecules [29,30]. EED proteins are crucial epigenetic regulators, and several published reports indicated that EED regulation depended on multiple context-sensitive *Pc*G complexes rather than gene dosage [15,17,18]. In addition, EED protein content has been found to vary among cell types [17]. Our results on EED-to-Gag stoichiometry in HIV-1 producer HEK-293T cells did not allow us to assess whether the biological effects of EED on HIV-1 replication were due to (i) a gene dosage effect or (ii) a wrong balance between EED isoforms and EED-containing complexes, e.g. EED3 + EED4 versus EED1 + EED2, or (iii) both. However, the observation that EED3 and EED4 isoforms preferentially localized in PRC3 complexes [23], and that HEK-293 epithelial cells had a high content of PRC1 and PRC2 proteins, compared to other epithelial cells such as HeLa [17], would be in favor of the second hypothesis.

Some clue to the cellular mechanism(s) of EED-mediated negative effect on virus yields was provided by our EM analysis of pTracer-EED- and pNL4-3Luc(R-E-)-cotransfected cells: cytoplasmic clusters of nuclear pore complexes in association with filament bundles reminiscent of annulate lamellae were observed in numbers in EED-expressing cells, and not in control cells (Fig. 10). Neoformation, ectopic localization or aberrant assembly of annulate lamellae and nuclear pore complexes (NPC) have already been described, in particular in malignant cells [37,38], fertilization-arrested human oocytes [39], certain virus-infected cells [40], and cells overexpressing the nuclear envelope pore membrane protein POM121 [41]. We hypothesized that exogenous EED3/4 mimicked the POM121 effect of recruitment of NPC proteins in ectopic nucleation sites [41] and provoked the aberrant assembly of NPC substructures within the cytoplasm, resulting in a sequestration or/and mistrafficking of viral genomic RNA (gRNA) or gRNA/Gag complexes and their failure to reach the virion assembly sites [42,43]. Our experimental results and hypothesis were compatible with the properties of EED proteins which shuttled between the nuclear and plasma membrane compartments [8], and interacted with NPC [12]. Thus, EED would be a restriction factor interfering with HIV-1 replication mostly at the level of virion production, and via different and nonexclusive mechanisms. Due to its interference with gRNA trafficking, EED would have an indirect negative impact on genome packaging and virus assembly. The negative effect of EED on genome packaging and virus assembly could also be mediated by interactions of EED with genome ends [13], and viral proteins MA and/or IN [11,12].

WTNef and NefG2A, but not the NefΔ57 mutant nor the lipid raft-targeted fusion LAT-Nef, restored virus production and infectivity to levels observed in the absence of EED (Fig. 6 and 7). This indicated that the EED-counteracting activity of Nef did not depend on its N-myristoylation and its virus packaging (abolished in NefG2A), but required its N-terminal domain, deleted from the NefΔ57 mutant. This confirmed the mapping of the EED-binding site to residues 16–35 in the N-terminal domain of Nef, although a second EED-binding region has been identified in the C-terminal domain [13]. This region overlapped the ED/EE motif identified as the v-ATPase binding site at position 174–175 in Nef, and was essential for plasma membrane recruitment of EED [13]. However, our experimental data with the NefΔ57 mutant suggested that the C-terminal EED-binding domain of Nef alone was not sufficient to reverse the negative effect of EED on virus yields. The fusion protein mutant LAT_AA_Nef, which lacked the lipid raft targeting function [26], showed the same phenotype as WTNef and NefG2A in terms of EED antagonistic effect (Fig. 7 and 8). This implied that the subset of Nef molecules localized in the lipid rafts did not contribute to the EED counteracting effect, and that the cellular compartment in which Nef bound and sequestered EED was different from the lipid rafts.

Interestingly, the WTNef-mediated positive effect on infectivity was more pronounced when vectors were produced in the presence of EED3/4, and was associated with a slight but consistent higher mean genome content per particle (Fig. 6). This cooperative effect between Nef and EED suggested the involvement of other cellular compartments or/and factors in virus production and infectivity. A recent study has shown the facilitation of HIV-1 egress mediated by Nef-AIP1 interactions, via their positive effect on multivesicular body (MVB) proliferation [46]. Alternatively, EED-Nef complexes might trap cellular factor(s) which negatively interfere(s) with virus assembly and viral genome incorporation, depleting them from the assembly sites. Nef might also compete for EED binding with certain cellular protein(s) which positively affect(s) the virus egress. In the two latter hypotheses, plasma membrane integrins, identified as partners of EED [8], might represent good candidates, since integrins are connected to tetraspanin-enriched microdomains (TEMs), and since TEMs represent potential gateways for HIV-1 egress [47]. The nature and mechanism of the Nef-mediated EED relocation are presently under investigation in other cells than the HEK-293T cell line.

## Methods

### DNA constructs

#### EED

For simultaneous expression of wild type (WT) EED3 and EED4 proteins in mammalian cells, the *eed *gene sequence from M95 to R535, as aligned with the mouse EED sequence of 535 residues [7], with a stop codon after the C-terminal residue (EED441 ; [12]), was cloned into the *EcoR *I and *Not *I sites of the expression plasmid pTracer™-EF/Bsd (version B ; Invitrogen). pTracer™-EF/Bsd was chosen to provide GFP protein for visual detection of transfected cells, as well as two C-terminal tags, V5 epitope and oligo-histidine. However, since the C-terminal extension of 30 residues could alter the biological properties of EED in one or the other way, we introduced a stop codon after the C-terminus of EED in pTracer-EED, to obtain untagged EED3/4 isoforms. The resulting plasmid was abbreviated pTracer-EED, and the control empty plasmid was referred to as pTracer-Emp. Plasmid pTracer-EED394 harbored the EED mutant ST394AI, defective in HIV-1 MA binding [11]. Plasmid pcDNA3-EED441 consisted of the EED441-coding sequence cloned into pcDNA3.

#### Nef

Plasmids expressing the different Nef proteins were obtained from D. Rekosh and M.-L. Hammarskjöld (University of Virginia at Charlottesville). WTNef was expressed using pHR1405 (pCMV-*nef *plasmid), which contained the full-length WT *nef *sequence from pNL4-3 under the control of the simian cytomegalovirus (CMV) IE94 gene promoter-enhancer [48], pHR1864 expressed the G-to-A substitution mutant (NefG2A), and pHR1871 the N-terminal deletion mutant NefΔ57 corresponding to the Nef core. Plasmids pHR2458 and pHR2462 encoded LAT and LAT mutant fusion proteins LAT-Nef and LAT_AA_Nef, respectively [26].

#### HIV-1-Luc vector

Plasmid phCMV-G encoded the vesicular stomatitis virus glycoprotein G under the control of the human CMV promoter with rabbit β-globin intron II and polyadenylation sequence [49]. Plasmid pNL4-3Luc(R-E-) [50] carried the firefly luciferase reporter gene (*luc*) in lieu of the deleted *nef *gene, and two frame-shift mutations in *vpr *and *env *genes.

### Cells and cell fractionation

Human embryonic kidney cells HEK-293T were grown in Dulbecco's modified glutamine-containing Eagle medium (Gibco), supplemented with antibiotics and 10 % fetal calf serum. For cell fractionation, 293T cells were lysed by three cycles of freezing and thawing in Tris-buffered saline (TBS), and lysates centrifuged at 8,000 × *g *for 10 min at 4°C. The supernatant (C1) was spared, and the first pellet (P1) was resuspended in TBS and subjected to a second round of centrifugation in the same conditions, giving supernatant C2 and intermediate pellet P2. Supernatants C1 and C2, corresponding to the cytosolic fraction, were pooled and abbreviated (C). Pellet P2 was then resuspended in TBS containing 0.5 % Triton X-100 for 30 min at 37°C, and centrifuged at 8,000 × *g *for 10 min at 4°C. The supernatant corresponded to Triton-soluble membrane fraction (M) comprising lipid rafts and non-rafts domains, and the final pellet (P) contained nuclei and cell organelles. For isolation of lipid rafts, cell lysates (ca. 1 ml) in HNE buffer (150 mM NaCl, 1 mM Na_2_EDTA, 25 mM HEPES, pH 6.5) containing a protease inhibitor cocktail (Boehringer) and 1 % Triton X-100 were mixed with an equal volume of 80 % sucrose, and overlaid with two layers of sucrose solution in HNE, 30 % (6 ml) and 5 % sucrose (4 ml), respectively. Samples were subjected to ultracentrifugation of flotation in a Beckman SW41 rotor for 18 h at 39 krpm and 4°C [51]. Anti-CD55 polyclonal antibody (H-319 ; Santa Cruz Biotechnology, Inc.) was used to detect the lipid rafts marker CD55.

### Gel electrophoresis, antibodies and immunoblotting

Polyacrylamide gel electrophoresis of SDS-denatured protein samples (SDS-PAGE), and immunoblotting analysis have been previously described [52,53]. Anti-HIV-1 Gag protein and anti-EED441-H6 protein rabbit antisera were laboratory-made [12,54]. Mouse monoclonal antibody (mAb) anti-CAp24 (Epiclone #5001) was purchased from Cylex Inc. (Columbia, MD), and anti-EED mAb (clone M26) was obtained from A. Otte (BioCentrum, Amsterdam). M26 has been raised against the N-terminal domain of the EED molecule, comprising of residues 95–174 [55]. Anti-HIV-1 reverse transcriptase (RT) mAb (clone 8C4D7; IgG1) was purchased from Intracell (Issaquah, WA). MAb anti-HIV-1 Nef C-terminal domain was obtained from Transgene SA (MATG-0020 ; Strasbourg, France) and had its epitope mapped to residues 161–175 [56]. MAb anti-VSV-G glycoprotein (clone P5D4) was purchased from Sigma. When needed, His-tagged proteins were detected using monoclonal anti-HisTag antibody (Qiagen) specific for N-, C-terminal and internal histidine clusters. Phosphatase-labeled anti-rabbit or mouse IgG conjugates were purchased from Sigma. For luminograms, chemiluminescent peroxidase substrate Supersignal™ (Pierce) was used. Immunological quantification of membrane-transferred proteins was performed by radio-immunoblotting [54], using ^35^SLR-labeled anti-rabbit or anti-mouse IgG antibody (Amersham Biosciences; 2,000 Ci/mmol; 10 μCi per 100 cm^2 ^membrane). Autoradiograms were scanned and quantitated by densitometric analysis, or alternatively, protein bands were excised from blots and radioactivity measured in a liquid scintillation spectrometer (Beckman LS-6500). Quantification of exogenous proteins in lysates from transfected cells was performed by scanning Coomassie Blue-stained SDS-PAGE gels. Protein content in EED or Pr55Gag bands was estimated by comparison with a range of BSA concentrations, after subtraction of the background signal from control cells.

### Production of HIV-Luc vector

VSV-G-pseudotyped HIV-1 competent for a single round of replication and carrying the *luc *gene was produced by cotransfection of 293T cells (producer cells) with phCMV-G and pNL4-3Luc(R-E-) at 10 μg each per 7 × 10^6 ^to 10 × 10^6 ^cells in standard experiments, with or without pTracer-EED or pTracer-Emp, using lipofectamine™ and Plus™ Reagent (Invitrogen) as transfecting agents. The cell culture supernatant containing the VSV-G pseudotyped HIV (abbreviated HIV-Luc) was collected at 48 h posttransfection, aliquoted and used for the infection of recipient 293T cells. HIV-Luc titers were determined by CAp24-antigen enzyme-immunoassay (VIDAS^® ^HIV P24 II test ; bioMérieux^® ^SA, Marcy-l'Etoile, France ; [57]), and expressed as CAp24 concentration (in pg/ml). Genomic RNA content of virions or cells was determined using an automated ribonucleic acid isolation technique [58,59] adapted to the NucliSens EasyMag™ Extractor (bioMérieux^® ^SA), and a real-time, isothermic gene amplification method (nucleic acid sequence based amplification or NASBA^® ^; [60]) with the NucliSens^®^EasyQ HIV-1 v1.1 kit (NucliSens^®^EasyQ platform; bioMérieux^® ^SA). Results were expressed as the number of genome copies/10^6 ^cells for cell lysates, and genome copies/ml for cell culture supernatants containing virus particles.

### Isolation of HIV-Luc virions

Two different methods of purification were used. *(i) Isopycnic gradient centrifugation*[54]. Virus particles released in the extracellular medium were analyzed by ultracentrifugation in sucrose-D_2_O gradients. Linear gradients (10-ml total volume, 30–50 %, w:v) were centrifuged for 18 h at 28 krpm in a Beckman SW41 rotor. The 50 % sucrose solution was made in D_2_O buffered to pH 7.2 with NaOH, and the 30 % sucrose solution was made in 10 mM Tris-HCl, pH 7.2, 150 mM NaCl, 5.7 mM Na_2_EDTA. Aliquots of 0.4 ml were collected from the top. *(ii) Sucrose-step gradient centrifugation *[61]. Cell culture supernatants were clarified by low-speed centrifugation, and virions recovered by pelleting through a sucrose cushion (20% in phosphate-buffered saline) at 30 krpm for 1 h at 15°C in a Kontron TST55.5 rotor.

### Functional assays for EED

#### (i) Effect on incoming virions

Single-round replication assays of VSV-G-pseudotyped HIV-Luc were performed in the absence (pTracer-Emp) or presence of exogenous EED3/4 proteins (pTracer-EED). Aliquots of recipient 293T cells were transfected with equal amounts of pTracer-EED or pTracer-Emp (1 μg/10^6 ^cells), or with plasmid inputs varying from 0 to 5 μg/10^6 ^cells. 24 h later, they were infected with HIV-Luc vector, at multiplicities of infection ranging from 20 to 80 ng CAp24 per 10^6 ^cells. Luciferase activity was measured at different times post-infection (pi) as previously described [62].

#### (ii) Effect on virus production

293T cells (2 × 10^6 ^cells) were cotransfected with 2 μg each of pNL4-3Luc(R-E-), phCMV-G, pTracer-EED (or pTracer-Emp), with or without Nef-expressing plasmid. Culture supernatants were collected at different times posttransfection, and assayed for exogenous RT activity [63,64], CAp24 titer, genomic RNA level and infectivity measured by luciferase assays on naive recipient 293T cells, as described above.

### RNA interference (siRNA)

Expression of short interfering RNA (siRNA) in 293T cells was performed using the pSUPER plasmid [65]. The insert in pSUPER (a gift from Dr. R. Agami [65]) was designed to express a 19 nt-long RNA sequence (AGCACTATGTTGGCCATGG) that has been identified as the most efficient oligonucleotide to target EED [66]. The resulting plasmid was referred to as pSUPER-i-EED. Cells were transfected with constant amounts (3 μg/10^6^cells) of a mixture of pSUPER + pSUPER-i-EED at various ratios of each plasmid, and 24 h later the cells were transfected with with pNL4-3Luc(R-E-). Virions were pelleted at 48 h posttransfection, and virus yields determined by viral genomic RNA level and CAp24 immunoassays.

### Cellular imaging

#### Confocal immunofluorescence microscopy

Cell monolayers were harvested at 48 h posttransfection, fixed with 2 % paraformaldehyde in phosphate buffered saline (PBS) and permeabilized in 0.2 % Triton X100 in PBS. Cells were blocked with 1% BSA in PBS (PBS-BSA), and reacted with rabbit anti-EED antibody (laboratory-made ; 1:200 in PBS-BSA) and Alexa Fluor^® ^488-labeled goat anti-rabbit IgG (Molecular Probes, Invitrogen), or mAb anti-Nef (USBiological H6004-15C ; epitope aa168-174 ; 1:200 in PBS-BSA) and Alexa Fluor^® ^633-labeled goat anti-mouse IgG antibody (Molecular Probes, Invitrogen). Samples were postincubated with DAPI and mounted on slides. Observations by confocal fluorescence microscopy were performed using a Leica TCS SP2 confocal microscope.

#### Electron microscopy (EM)

Cells harvested at 48 h posttransfection were pelleted, fixed with 2.5% glutaraldehyde in 0.1 M phosphate buffer, pH 7.5, post-fixed with osmium tetroxide (2 % in H_2_0) and treated with 0.5% tannic acid solution in H_2_0. The specimens were dehydrated and embedded in Epon (Epon-812; Fulham, Latham, NY). Sections were stained with 2.6 % alkaline lead citrate and 0.5 % uranyl acetate in 50 % ethanol, and post-stained with 0.5% uranyl acetate solution in H_2_0 [12,67]. Specimens were examined under a Jeol 1200-EX electron microscope, equipped with a MegaView II high resolution TEM camera and a Soft Imaging System of analysis (Eloïse, Roissy, France). About 150 independent cell sections (10 to 12 separate fields of 10 to 15 cells each) were examined under the electron microscope.

## Competing interests

The author(s) declare that they have no competing interests.

## Authors' contributions

DR performed most of the laboratory work, and PB performed the EM analyses. PB, JCT, JLD and SSH conceived the strategies and designed the experiments. PA contributed to discussion and data analysis. PB and JLD wrote the manuscript. All authors read and approved the final manuscript.

## Supplementary Material

Additional file 1 Electron microscopic analysis of 293T cells cotransfected with pNL4-3Luc(R-E-) and pTracer-EED. The ultrathin section of this cell, harvested at 48 h posttransfection, shows clusters of ectopic nuclear pore complexes (NPC) within the cytoplasm, besides NPC associated with the nuclear envelope. Click here for file

Additional file 2 Electron microscopic analysis of 293T cells cotransfected with pNL4-3Luc(R-E-) and pTracer-EED. The ultrathin section of this cell, harvested at 48 h posttransfection, shows clusters of ectopic nuclear pore complexes (NPC) associated with bundles of cytoplasmic filaments. Click here for file
