# Supplementary figures and images for: Human Polycomb group EED protein negatively affects HIV-1 assembly and release
Source: Retrovirology. 2007 Jun 4;4:37. doi: 10.1186/1742-4690-4-37 (PMC1899515; doi:10.1186/1742-4690-4-37)

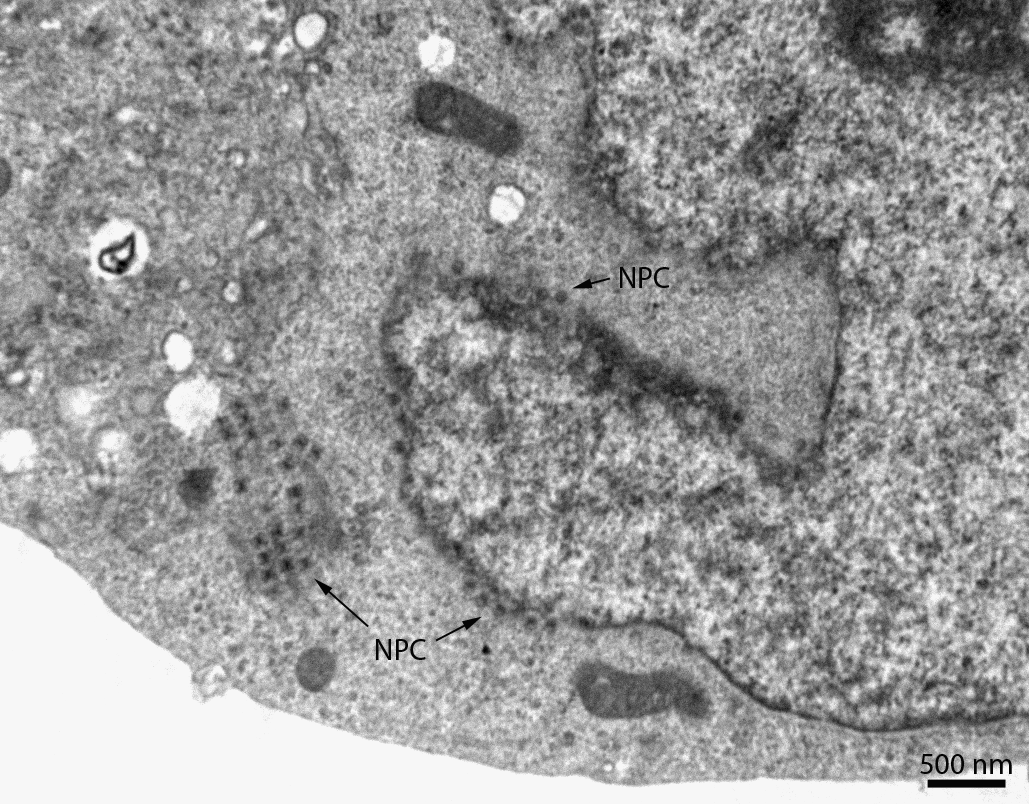

Supplement: Additional file 1 — Electron microscopic analysis of 293T cells cotransfected with pNL4-3Luc(R-E-) and pTracer-EED. The ultrathin section of this cell, harvested at 48 h posttransfection, shows clusters of ectopic nuclear pore complexes (NPC) within the cytoplasm, besides NPC associated with the nuclear envelope. [file 1742-4690-4-37-S1.tiff]

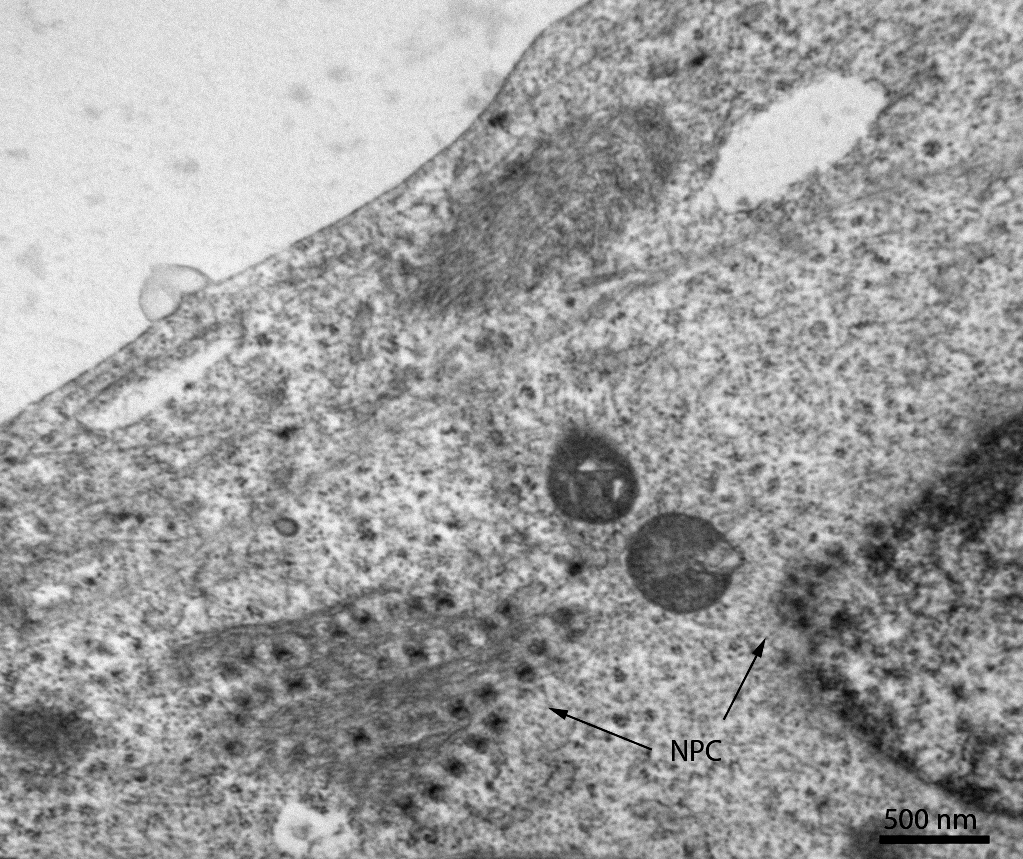

Supplement: Additional file 2 — Electron microscopic analysis of 293T cells cotransfected with pNL4-3Luc(R-E-) and pTracer-EED. The ultrathin section of this cell, harvested at 48 h posttransfection, shows clusters of ectopic nuclear pore complexes (NPC) associated with bundles of cytoplasmic filaments. [file 1742-4690-4-37-S2.tiff]
